# Experience in Dentistry

**Published:** 1897-06

**Authors:** H. C. Rockwell

**Affiliations:** Benton Harbor, Mich.


### Experience in Dentistry.

Read before the Dental Society of Southwestern Michigan at Kalamazoo, April 14th, 1897.

BY H. C. ROCKWELL, D.D.S., BENTON HARBOR, MICH.

When, a very young man, I commenced the study of dentistry, experience was considered more important than any amount of dental education.

A course in college was looked upon more as an ornamental adjunct, to put the fancy touches upon one who had already learned the business, than as a necessity, and the dental student dug his way through the plaster and sand of the laboratory to a practical knowledge of his profession.

The instruction was something after the kindergarten method, a little after the fashion first promulgated by the celebrated "Wackford Squeers," of "Dortheboy's Hall."

As you may remember, "This is the first class in English, Spelling and Philosophy," said Squeers. "Now then, the first boy." "Please Sir, he is cleaning the back parlor window." "So he is, to he sure," rejoined Squeers. "We go upon the practical mode of teaching the regular education system. C-l-e-a-n clean—verb—active, to make bright—to scour. W-i-n win, d-e-r der, winder, a casement. When the boy knows this out of book he goes and does it. Now the second boy." "Please Sir, he's weeding the garden." "So he is. B-o-t bot, t-i-n tin, bottin, n-e-y ney, Bottinney—a knowledge of plants, and when the boy learns this he goes and knows 'em." "That's our system. How do you like it?" "It's a very useful one at all events," rejoined his auditor.

All colleges required three years tuition in a dental college as a prerequisite to graduation. In the office where I begun, there was plenty of work for all hands. In the first place, there was the "old man," so termed by the rest of us; then the senior student, in his third year, the sort of "middle sized" student, so to speak, in his second year, and myself, just commencing.

There was a sort of tacit understanding in that office, which had come to be the unwritten law, that the members of the profession therein employed needed rest exactly in proportion to their standing and dignity. The "old man" never did any work that the senior student could do as well as he could; the senior student never interfered with the rights of the junior in that regard, and the junior, having the example of both of his superiors before his eyes, never failed to impress upon the freshman that experience in dentistry was of the utmost value, and seldom failed to see that the freshman got it, especially if that experience was of a nature somewhat disagreeable to anybody else. The "old man" waited on the ladies, and his oldest and best patients, and only stooped so far as to put in the gold fillings; the senior prepared the cavities, put in the amalgam fillings, and as many of the gold ones as the patients would let him; the junior prepared a few cavities, took impressions, and set up the teeth; the freshman "did the rest."

Now in an office where all hands were expected to begin work at seven o'clock and keep at it as long as we could see, and the freshman, not only to do the vulcanizing in the evening, but to be up early and have the office cleaned up before seven, you may see that the kind of experience and education spoken of by Wackford Squeers, aforesaid, was not lacking, and that the boy had not only gone and done it once, but so many times that he could do it practically, if not theoretically, well.

The next year I was the junior, and made the freshman polish the plates and do the dirty work while I set up teeth, took impressions and prepared cavities, and so on up the ladder of progress in the office until, when I came to leave and start for myself, there was hardly an ordinary operation in dentistry but I had performed a hundred times.

I thought many times, during my last year in the office, and after I had graduated at a dental college, that it was a little tough to work hard day after day and see the "old man" "rake in the ducats," but many has been the time since I have blessed the facility and rapidity which I acquired under the old system.

I know that nowadays the student many times begins at the other end—he graduates first and gets his experience afterward; and many of our colleges rather deprecate the private tutor for fear that the student may acquire habits and tricks difficult to teach him out of.

This may be correct to a certain extent, but if I had been obliged to acquire the experience which I obtained in my three years under a tutor, it would have taken me ten, with the amount of practice I was able to get together, in my early start for myself. It is said that we learn more by our failures than by our successes if that be true you can learn pretty fast when first beginning in dentistry, especially if you have three seniors over you in the office to find fault and inquire "why in thunder" didn't you do it the right way. Under the new way a student is supposed to perform each operation once during his college course, and to perform that operation theoretically correct. Under the old way he performed each operation a good many times, if not theoretically correct, it had to be pretty practically good or the "old man" "climbed onto his frame" for carelessness.

I sometimes think that we have gone too far in the new direction—that it would be easier to bring up a good many bad habits and practices to a correct theory; but no amount of theory can give that facility, that manual dexterity, and that rapidity which is so important to the dentist. I may be regarded by many as heretical. If I had a tooth to fill myself, or in fact any operation in dentistry to have performed, I would take my chances with a student who had been brought up under the old kindergarten method, and had never seen a college, rather than trust the valedictorian of the college class, if he had never worked in an office and only done the work required during his college term.

Now I don't want it understood that I underrate the value and necessity of the college course, but I do know that I could have performed almost any operation fairly well by what I learned in the office. I fear it would have been many a long year before I could have done as well by what I learned at the college.

Experience evolves after a while the correct theory; theory will never bring manipulative ability, certainty and speed. The boy must, as Squeers said, "go and do it" not only once, but many times. Experience is of great value—education also.

What the progressive and enlightened dentist should have is *both*.

Like the Irishman who, at the hotel table, was asked by the waiter whether he would take pie or pudding, replied, "Yis." "Well," said the waiter, "I said would you take pie or pudding." "Well," said our Irishman, "be jabers, didn't I say yis?" When asked if we will take education or experience, like him, we should say "*yes*."

I know we old fellows—I mean Dr. ———, Dr. ———, Dr. ——— and myself, are very apt to think we have seen a whole lot during our many years of plugging, and there is a dangerous tendency toward thinking we have absorbed pretty much all there is of value, or is worth knowing in the profession. We "work our jaws" at dental society meetings and elsewhere, something after the manner that the prize fighters, and Chinese warriors do, so as to make younger ones think we know it all, but don't let Dr. ——— know I gave it away—the fact of the case is, we don't.

New discoveries, new theories, new methods—one "doth tread upon another's heels, so fast they follow" in this, our chosen profession. Any man who depends on either the education or experience already acquired will soon be far in the rear of the "advance guard." Experience enables many of us to steer clear of the numberless old fakes which are every year brought forward as new and valuable inventions. We should beware, lest it leads us to neglect some that are really new and valuable.

Experience should teach us to "Be not the first by whom the new is tried, nor yet the last to lay the old aside." Experience should teach us that the time spent in attendance on our Dental Society is of more value than if spent in the office—that "in union there is strength." Not only strength and standing in the community, but the renewed vigor born of increased knowledge of our professional work and of one another.

If we stay in our offices and rely on the statements of the shopping patient, we are liable to think that the fellow across the way is, to put it mildly, "A curious little cuss," as Artemus Ward said his monkey was. When we get together in our society, experience tells us that it was the troublesome patient that was not only "curious," but a confoundedly mean cuss after all.

Experience should teach us that money is by no means the only, or even the greatest, good to be sought in a professional life—that, as the old Scotch Dominie told his miserly parishoner, "Ye can't carry your gowd wi' ye when ye die, if ye did may be twould melt."

Experience should teach us that whether or not we believe in the various codes of ethics laid down and more or less observed by the members of many of our societies, we should all believe in, and try and live up to, that greater and better code, wisely laid down for our guidance so many years ago by the great teacher. "Do ye unto others as ye would that they should do unto you," and living up to this code, experience should teach us that it is far better to try and educate the great number who need our professional services, and extend our practice and usefulness in that way, than to waste our ingenuity in efforts to take patients from our brother across the way.

Experience should teach us that ours is a liberal profession; that we distinguish it ourselves as we become liberal, broad guage men.

And finally, my brothers, experience should teach us, every one, to fill his alotted place in life in such a way that when his summons comes, as come it must to each and all, mayhap as unexpected, swift and sudden as it came to our friend and brother, Dr. Ray, whether it comes while yet the eye is bright in youth and the form erect in the vigor of manhood, or when the shoulders are bowed with the weight of years, and upon the head has thickly fallen "that snow that never melts." Whenever and wherever it may be, those with whom he was associated may truthfully say, he was a professional gentleman—he was an honorable citizen—he was an honest man.

"On parent knees, a naked, new born child,
Weeping thou sats't, while all around thee smiled,
So live, that sinking in thy last, long sleep,
Calm thou may'st smile, while all around thee weep."

